# Radiotherapy for Hypopharyngeal Carcinoma: Analysis of Multi-institutional Data From Over 170 Patients

**DOI:** 10.7759/cureus.96995

**Published:** 2025-11-16

**Authors:** Hideya Yamazaki, Gen Suzuki, Aibe Norihiro, Takuya Kimoto, Koji Masui, Kanako Kawabata, Shinsuke Nagasawa, Yuki Yoshino, Shoko Hirano, Akito Asato, Satoshi Ikeda, Kei Yamada, Naohiro Kodani, Daisuke Shimizu

**Affiliations:** 1 Radiology, Kyoto Prefectural University of Medicine, Kyoto, JPN; 2 Radiation Oncology, Japanese Red Cross Society Kyoto Daiichi Hospital, Kyoto, JPN; 3 Radiation Oncology, Japanese Red Cross Society Kyoto Daini Hospital, Kyoto, JPN

**Keywords:** cancer of the head and neck, chemoradiotherapy, hypopharyngeal cancer, intensity modulated radiotherapy, radiotherapy

## Abstract

Aim

To examine the efficacy of radiotherapy (RT) for treating hypopharyngeal carcinoma (HPC), including the impact of transitioning from conventional three-dimensional conformal radiotherapy (3DCRT) to intensity-modulated radiotherapy (IMRT).

Materials and methods

We examined the outcomes of 171 patients with Stage I-IV HPC who were treated with definitive RT (87 with 3DCRT and 84 with IMRT) at three institutions between 2008 and 2024. The primary endpoint was overall survival (OS), and the secondary endpoints were local control (LC), locoregional control (LRC), progression-free survival (PFS), and toxicity.

Results

With a median follow-up time of 32 months (range, 2-127 months), the 2-year (2y) and 5-year (5y) OS rates were 83.9% (95% confidence interval [CI]: 77.1-88.9%) and 69.0% (95% CI: 58.8-77.1%), respectively. The 2y LC (5y), LRC, and PFS were 81.1% (74.1%), 78.1% (71.3%), and 64.3% (52.3%), respectively. The advanced T category was a statistically significant predictor of poor prognosis for both LC and LRC in the univariate analysis. Multivariate analyses found that N category (N0-1 vs. N2-3, hazard ratio = 1.7, 95% CI: 1.16-2.5, P = 0.0068) was a statistically significant predictor of OS. Five patients (2.8%) experienced late toxicities of grade ≥ 3. The transition from conventional 3DCRT to IMRT did not significantly alter the tumor control or toxicity profiles.

Conclusion

Definitive RT achieved favorable OS, LC, LRC, and PFS in patients with HPC, with acceptable toxicity. IMRT provides comparable efficacy and safety to 3DCRT for the treatment of HPC.

## Introduction

Hypopharyngeal carcinoma (HPC) is relatively rare, accounting for only 0.4% of new cancer cases worldwide in 2020 [1]. HPCs have a worse prognosis than other head and neck cancers because of their late presentation and greater propensity for nodal metastasis. Approximately 80% of cases are diagnosed as locally advanced disease upon presentation [2].

Although surgery has the potential to cure, it can induce substantial functional morbidity in speech and swallowing. Therefore, alternative organ-preserving treatment modalities, such as radiotherapy, particularly chemoradiotherapy-based approaches, should be considered. Upfront concurrent chemoradiotherapy (CCRT) is endorsed by both contemporary National Comprehensive Cancer Network and European Society for Medical Oncology guidelines as a well-established larynx preservation strategy, alongside induction chemotherapy (IC) [2,3].

Several new technologies have been introduced for the management of HPC in the 21st century. First, intensity-modulated radiotherapy (IMRT), a new RT technique, was introduced and became widespread [4-6]. IMRT enables highly conformal dose distributions by modulating the intensity of multiple radiation beams. This allows the prescribed dose to be delivered precisely to complex tumor geometries. Therefore, IMRT could reduce toxicity and improve tumor control by targeting tumors and organs at risk more effectively. Next came the new systemic therapies of IC and bio radiotherapy (BRT), which were introduced alongside standard triweekly CDDP CCRT [7-10]. However, the impact of these new technologies is not fully understood. Therefore, this study was conducted to analyze the efficacy of radiotherapy (RT) for HPC and examine the roles of newly installed technologies in a multi-institutional database comprising more than 170 patients.

## Materials and methods

Patients and methods

We retrospectively examined the data of patients with HPC who received RT for HPC between 2008 and 2023 at the Department of Radiology, Kyoto Prefectural University of Medicine, Department of Radiation Oncology, Japanese Red Cross Society Kyoto Daiichi Hospital, and Japanese Red Cross Society Kyoto Daini Hospital. We included patients with histologically confirmed HPC (squamous-cell carcinoma (hypopharynx) with clinical T1-4N0-3M0 disease and available and accessible data on the T, N, and M classifications according to the NCCN risk classification (Union for International Cancer Control, 7th Edition)). We excluded patients with distant metastases at diagnosis and RT discontinuation before the planned dose.

RT was generally performed according to the guidelines and with a median prescribed dose of 70 Gy (range: 56-73 Gy) in 35 fractions (range: 16-42 fractions) [2,3,11].

In the conventional three-dimensional conformal radiotherapy (3DCRT) group, 40 Gy was generally delivered to the entire neck, including the prophylactic lymph node area [11]. An additional 26-30 Gy boost was delivered to the gross tumor and involved lymph nodes using the cone-down technique to spare the spinal cord from radiation exposure. The major 3DCRT schedules were 70 Gy in 35 fractions (n = 67) and 60 Gy in 30 fractions (n = 11).

In the IMRT group, the main treatment schedule involved delivering 70 Gy over seven weeks to the primary and node planning target volume (PTV), while 40 Gy was delivered to the initial PTV, including the prophylactic lymph node area. The prescribed IMRT dose was calculated based on D95 (95% of the target volume receives the prescribed dose) for the primary and node PTVs, while the dose for the initial node PTV was calculated based on. RT was administered at 2 Gy per fraction, once a day. Gross tumor volume (GTV) was based on clinical, endoscopic, and radiological findings on CT, MRI, and PET/CT. The clinical target volume (CTV) was generated by adding a 5-10 mm margin from the GTV, including the primary tumor and affected lymph nodes. The PTV was generated by adding a 5-10 mm margin from the CTV. All treatment plans were designed using a linear accelerator equipped with multi-leaf collimators or helical tomotherapy. The major IMRT schedules were 70 Gy in 35 fractions (n = 40) and 68.6 Gy in 33 fractions (n = 38).

For the target lesion, we prescribed a dose to the isocenter or center of PTV in 3DCRT, and used the following parameters: PTV D98% goal: >93% (acceptable: >90%); D95% goal: 100% (acceptable: >98%) in IMRT. For OARs: i.e., dose-volume criteria for organs at risk were “i.e., spinal cord max dose 45 Gy, parotid mean dose 26 Gy in 3DCRT, and parotid Dmean, goal <24 Gy, (acceptable <30 Gy) at least one of them (left and right), spinal cord PRV (= spinal cord+3 mm) D1cc Goal <46 Gy, (acceptable < 50 Gy) in IMRT [3]”.

Systemic therapy included IC, CCRT, and BRT. The primary IC medication administered was a combination of docetaxel, cisplatin, and 5-fluorouracil (TPF therapy). TPF comprised docetaxel at 75 mg/m² on days 1, cisplatin 75 mg/m² on days 1 and 5-FU 750 mg/m² on days 1-5. This was administered in two-three cycles, each three weeks apart. IC was indicated for patients with a disease classification of ≥T3 or ≥N2b, for whom a larynx-sparing strategy could be considered. The main CCRT regimen comprised three cycles of cisplatin at 100 mg/m² at three-week intervals.

In BRT, cetuximab was administered at an initial dose of 400 mg/m² one week before the start of radiotherapy, followed by weekly doses of 250 mg/m² throughout the radiotherapy period. BRT is indicated for patients aged 75 years or older, or in cases where cisplatin is contraindicated due to renal impairment.

We analyzed overall survival (OS) as the primary endpoint and local control (LC) rate, locoregional control (LRC) rate, progression-free survival (PFS), and toxicity as secondary endpoints. We defined OS as the time between the start of treatment and the last follow-up date or death. LC and LRC of the tumor were defined as the absence of recurrence at the primary site (LC) and in the regional lymph node area (LRC). PFS was defined as the duration of freedom from any locoregional or distant recurrence or death. The time of the event was determined from the start of RT. Adverse events were classified according to the National Cancer Institute Common Terminology Criteria for Adverse Events, version 4.0. All patients included in the analysis provided written informed consent. This study was conducted in accordance with the principles of the Declaration of Helsinki and was approved by the Kyoto Prefectural University Institutional Review Board (ERB-C-3572) and the review boards of each of the hospitals involved.

Statistical analyses

The EZR stat package was used for statistical analyses [12]. Percentages were analyzed using Fisher's exact test for two groups and the chi-squared test for three or more groups. Comparisons were made using Student’s t-tests for normally distributed data and Mann-Whitney U-tests for skewed data. The Kaplan-Meier method was used to analyze OS, LC, LRC, and PFS, which were compared using the log-rank test. We used Cox’s proportional hazard model for the multivariate analysis for the primary endpoint (OS). The following variables were entered into the multivariate analysis: age, sex, T category (T1-2 vs. T3-4), N category (N0-1 vs. N2-3), use of systemic therapy, and RT technique (3DCRT vs. IMRT). Cut-off values were set at the median or mean values if not specified. p < 0.05 was considered to be statistically significant.

## Results

A total of 171 patients underwent radiotherapy for non-metastatic HPC between April 2008 and September 2024 at three institutions. The detailed characteristics of the patients, tumors, and treatments are shown in Table 1. The median age of all patients was 69 years (range, 43-90 years), and 86.0% of the patients were male.

**Table 1:** Patients’ characteristics in the total population. NOS: Not otherwise specified; IC: induction chemotherapy; CCRT: concurrent chemoradiotherapy; BRT: bio radiotherapy; 3DCRT: three-dimensional conformal radiotherapy, IMRT: intensity-modulated radiotherapy The total number of patients receiving each systemic therapy regimen does not equal the total number of patients, because several patients received more than one regimen.

Variables	Strata	No. (%) or Median (Range)
		(n=171)
Age	years	69.00 (43.00, 90.00)
Gender	F	24 (14.0)
	M	147 (86.0)
Subsite	NOS	3 ( 1.8)
	Post cricoid	14 ( 8.2)
	Piriform sinus	116 (67.8)
	Posterior wall	38 (22.2)
T	1	21 (12.3)
	2	87 (50.9)
	3	28 (16.4)
	4	35 (20.5)
N	0	59 (34.5)
	1	21 (12.3)
	2	82 (48.0)
	3	9 ( 5.3)
Stage	1	10 ( 5.8)
	2	40 (23.4)
	3	24 (14.0)
	4	97 (56.7)
Systemic therapy	No	38 (22.4)
	Yes	133 (77.8)
IC	Yes	72
CCRT	Yes	95
BRT	Yes	19
RT technique	3DCRT	87 (50.9)
	IMRT	84 (49.1)

**Figure 1:**
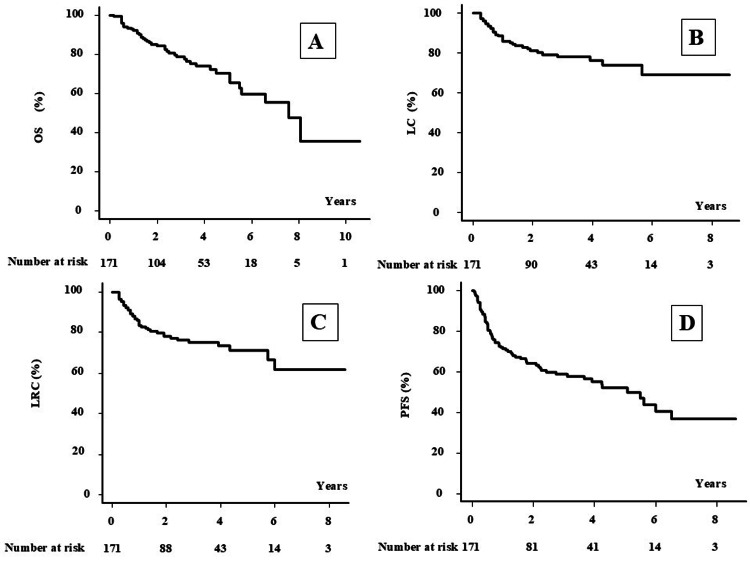
Overall survival, local control, locoregional control, and progression-free survival rates in the total population. A: Overall survival (OS), B: local control (LC), C: locoregional control (LRC), D: progression-free survival (PFS) rates in the total population.

**Figure 2:**
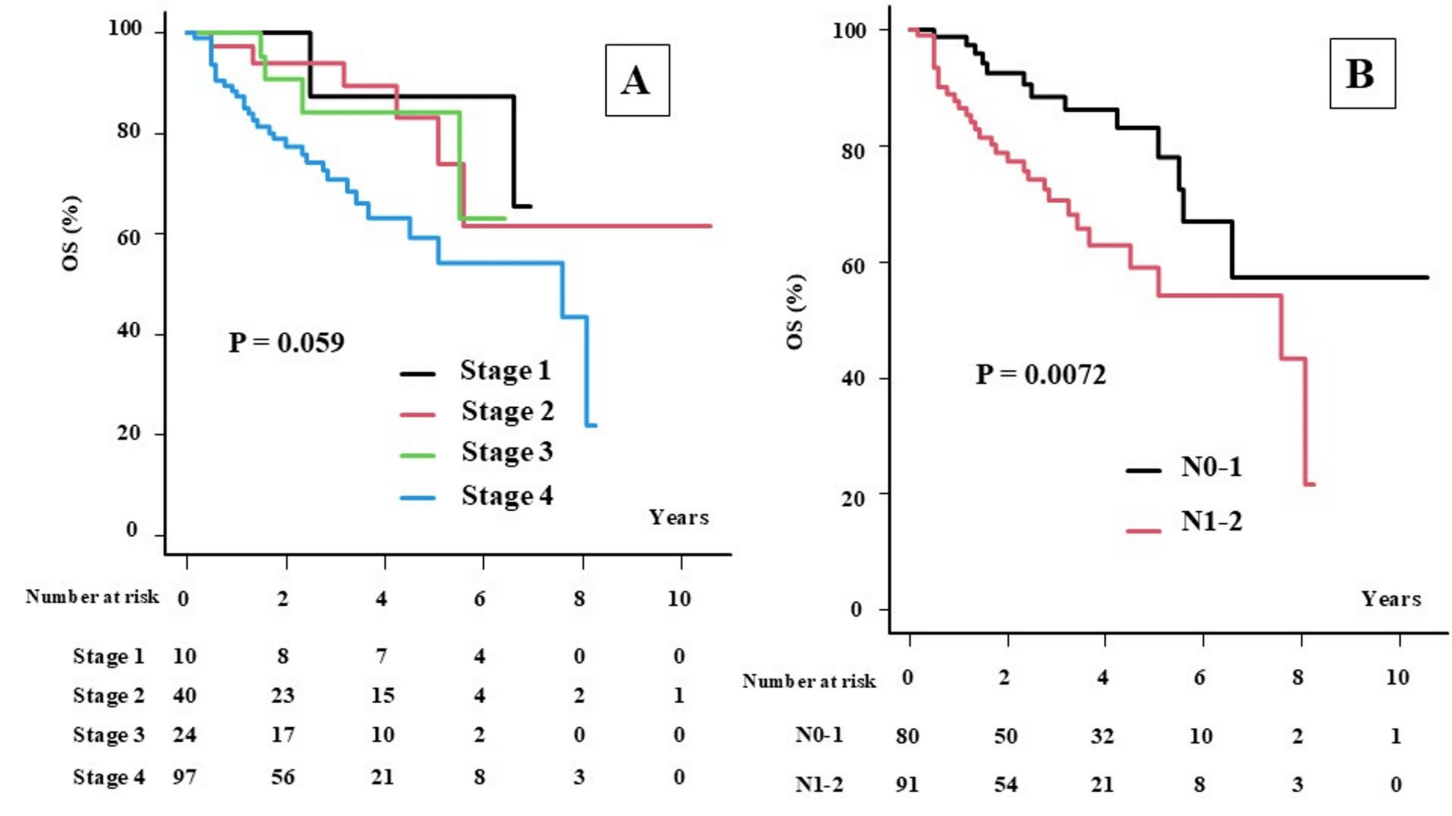
The overall survival rate according to stage classification and N category. A: Overall survival (OS) according to stage classification. B: Overall survival (OS) according to N category (N01 vs. N2-3).

The median follow-up time was 32 months (range, 2-127 months) for the total population and 51 months (range, 6-127 months) for survivors. At the final follow-up, 127 patients were alive, and 44 had died. The two-year OS (2yOS) and five-year OS (5yOS) were 84.4% (95% confidence interval (CI): 77.5-89.3%) and 70.6% (95% CI: 60.6-78.5%), respectively, for patients with HPC (Figure 1). By stage, the 2yOS (5yOS) was 100% (87.5%), 94.1% (83.3%), 90.9% (84.4%), and 77.5% (59.3%) for patients with stages I, II, III, and IV disease, respectively (P = 0.059; Figure 2A).

**Figure 3:**
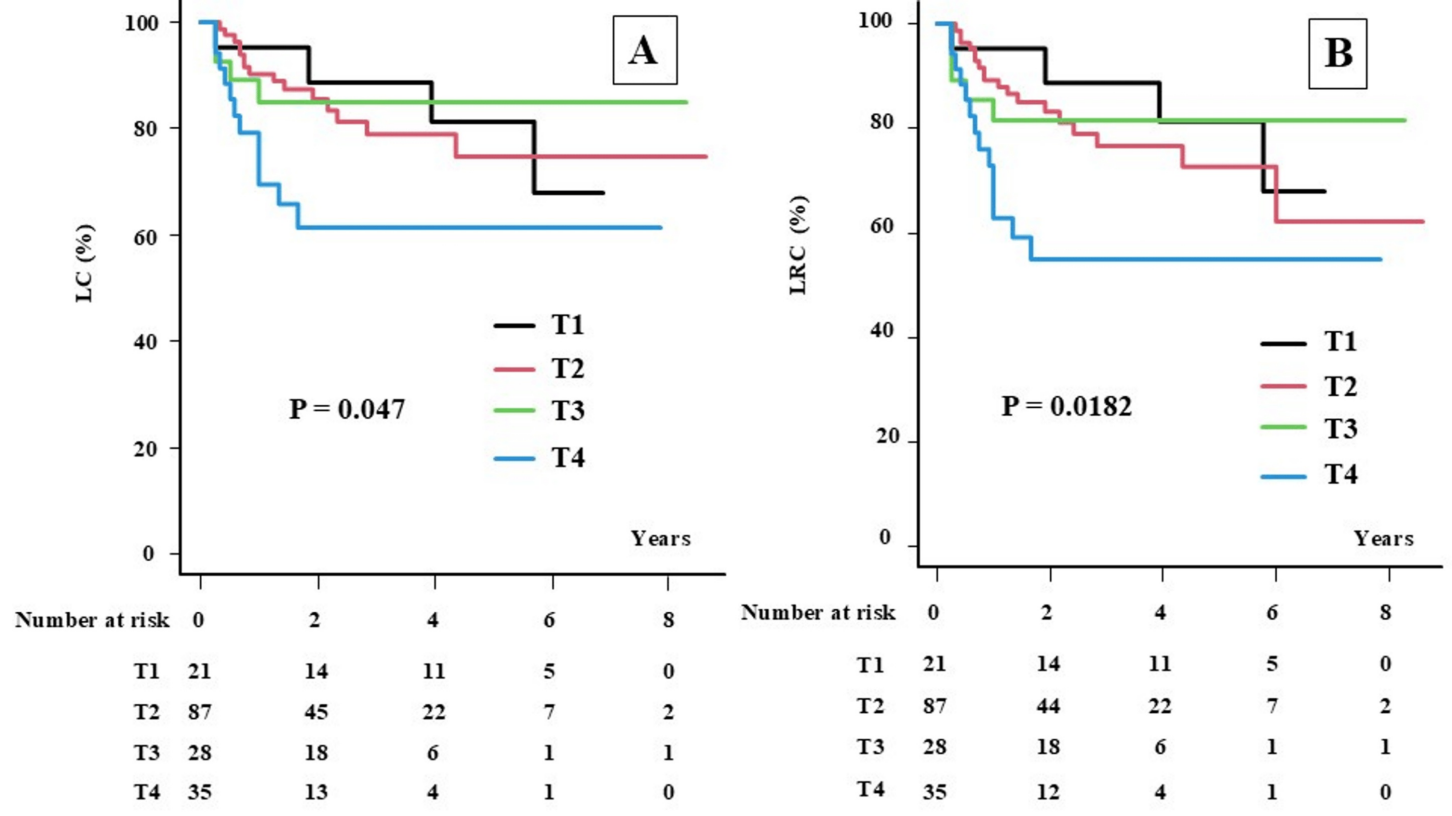
Local control and locoregional control rates according to T category. A: Local control (LC) rate according to T category, B: Locoregional control (LRC) rate according to T category.

The two-year OS (2yOS) and five-year OS (5yOS) were 84.4% (95% confidence interval (CI): 77.5-89.3%) and 70.6% (95% CI: 60.6-78.5%), respectively, for patients with HPC (Figure 1). By stage, the 2yOS (5yOS) was 100% (87.5%), 94.1% (83.3%), 90.9% (84.4%), and 77.5% (59.3%) for patients with stages I, II, III, and IV disease, respectively (P = 0.059; Figure 2a). The 2yLC (5yLC), 2yLRC (5yLRC), and 2yPFS (5yPFS) were 81.1% (74.1%), 78.1% (71.3%), and 64.3% (52.3%), respectively (Figures 1B-1D). Univariate analysis revealed that the T category was a statistically significant predictor of poor prognosis for both LC and LRC (Table 2 and Figure 3).

**Table 2:** Overall survival, local control, locoregional control, and progression-free survival rates according to patients, tumor, and treatments IC: induction chemotherapy; CCRT: concurrent chemoradiotherapy; BRT: bio radiotherapy; 3DCRT: three-dimensional conformal radiotherapy; IMRT: intensity-modulated radiotherapy; OS: overall survival; LC: local control; LRC: locoregional control; PFS: progression-free survival rate

	2-Year Survival Rate								
Factor	N	OS	P-value	LC	P-value	LRC	P-value	PFS	P-value
Age									
≤ 67 years	72	0.85	0.33	0.79	0.83	0.75	0.94	0.62	0.525
≥ 68 years	99	0.84		0.83		0.81		0.66	
Gender									
Female	24	0.86	0.61	0.70	0.09	0.65	0.08	0.57	0.143
Male	147	0.84		0.83		0.80		0.66	
Subsite									
Post cricoid	14	0.83		0.77		0.70		0.59	0.498
Piriform sinus	116	0.86		0.83		0.81		0.68	
Posterior wall	38	0.83		0.74		0.72		0.56	
NOS	3	0.67	0.80	1.00	0.46	1.00	0.41	0.67	
T category									
1	21	0.94	0.30	0.89	0.049	0.89	0.02	0.75	0.154
2	87	0.87		0.86		0.83		0.67	
3	28	0.86		0.85		0.82		0.70	
4	35	0.71		0.61		0.55		0.47	
N category									
0	59	0.96	0.06	0.83	0.80	0.83	0.42	0.77	0.198
1	21	0.85		0.86		0.86		0.71	
2	82	0.77		0.79		0.74		0.56	
3	9	0.78		0.74		0.59		0.44	
Stage									
1	10	1.00	0.06	1.00	0.14	1.00	0.13	0.90	0.151
2	40	0.94		0.74		0.74		0.67	
3	24	0.91		0.96		0.96		0.83	
4	97	0.78		0.78		0.72		0.56	
Systemic therapy									
No	38	0.86	0.39	0.88	0.64	0.85	0.57	0.72	0.783
Yes	133	0.84		0.79		0.76		0.62	
IC									
Yes	72	0.76		0.74		0.70		0.52	
CCRT									
Yes	95	0.86		0.78		0.74		0.61	
BRT									
Yes	19	0.89		0.90		0.90		0.79	
RT technique									
3DCRT	87	0.83	0.37	0.86	0.26	0.85		0.70	0.391
IMRT	84	0.85		0.77		0.72		0.58	

For each T category, the 2yLC (5yLC) was 88.9% (81.5%), 85.6% (74.9%), 85.0% (85.0%), and 61.4% (61.4%) for patients in the T1, T2, T3, and T4 categories, respectively (P = 0.0474, Figure 3A). The 2yLRC (5yLRC) was 88.9% (81.5%), 83.1% (72.6%), 81.6% (81.6%), and 55.0% (55.0%) for patients with T1, T2, T3, and T4 categories, respectively (P = 0.0182, Figure 3B).

Multivariate analyses found that the N category (N 0-1 vs 2-3, hazard ratio = 1.70, 95% CI: 1.16-2.50, P = 0.0068) was a statistically significant predictor for OS (Table 3).

**Table 3:** Multivariate analysis for predictive factor of OS. 3DCRT: three dimensional conformal radiotherapy, IMRT: intensity modulated radiotherapy

Variables	Strata	Hazard Ratio (95% Confidence Interval)	p-value
Age	≤ 67 vs ≥ 68 years	1.03 (0.53-2.00)	0.93
Gender	Female vs Male	0.97 (0.42-2.25)	0.94
Systemic therapy	Yes vs. No	0.55 (0.26-1.17)	0.12
T category	1-2 vs. 3-4	1.29 (0.65-2.53)	0.47
N category	0-1 vs. 2-3	1.70 (1.16-2.50)	0.0068
RT techniques	3DCRT vs. IMRT	0.60 (0.30-1.20)	0.15

The 2yOS (5yOS) was 92.5% (83%) and 77.3% (59%) for patients in the N0-1 and N2-3 categories, respectively (P = 0.0072, Figure 2B).

As systemic therapy was primarily administered for advanced disease, the efficacy of each regimen (IC, BT, CCRT, and IC followed with BR or CCRT) is shown in Table 4 according to stage classification.

**Table 4:** Overall survival and predisposing factors according to stage, systemic therapy, and radiotherapy technique. IC: induction chemotherapy; CCRT: concurrent chemoradiotherapy; BRT: bio radiotherapy; NA: not available; 3DCRT: three-dimensional conformal radiotherapy; IMRT: intensity-modulated radiotherapy The total number of patients receiving each regimen does not equal the sum because several patients received multiple regimens.

	2-year Overall Survival Rate								
Factor	N	Stage I	N	Stage II	N	Stage III	N	Stage IV	p-value
Systemic therapy									
No	7	1.00	18	0.94	2	0.50	11	0.72	0.0165
Yes	3	1.00	22	0.93	22	0.95	86	0.78	
IC									
Yes	1	1.00	5	1.00	10	0.89	56	0.71	
BRT									
Yes	0	NA	4	1.00	2	1.00	13	0.84	
CCRT									
Yes	3	1.00	17	0.91	19	0.94	56	0.82	
Upfront CCRT or BRT									
Yes	2	1.00	18	0.91	13	1.00	47	0.82	
IC followed with CCRT or BRT									
Yes	1	1.00	4	1.00	9	0.88	39	0.74	
RT technique									
3DCRT	9	1.00	26	0.92	10	0.94	42	0.70	0.092
IMRT	1	1.00	14	1.00	14	0.83	55	0.84	

**Figure 4:**
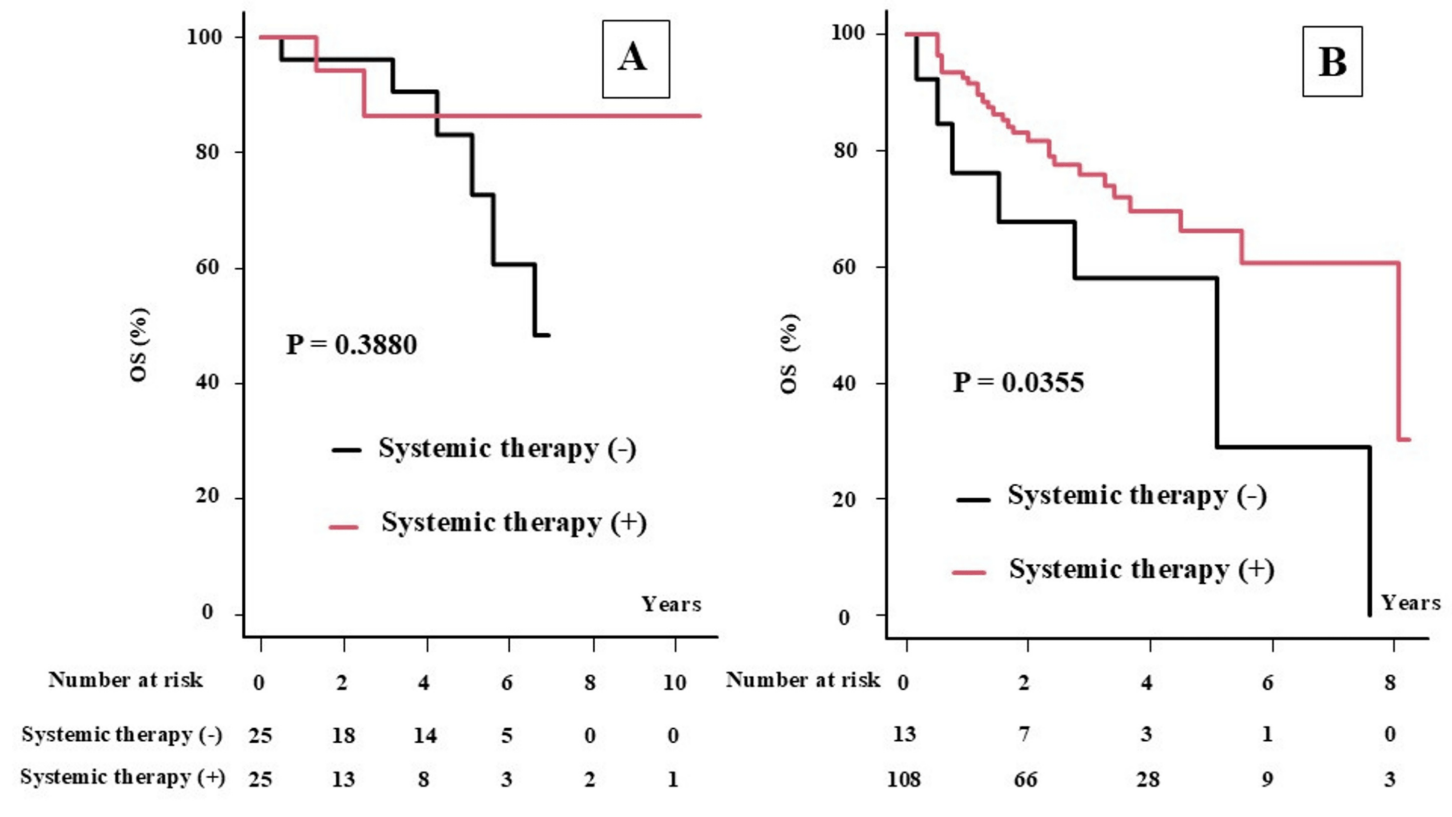
The overall survival rate according to stage classification and systemic therapy. A: Overall survival (OS) with or without systemic therapy in stage I-II disease. B: Overall survival (OS) with or without systemic therapy in stage III-IV disease.

Systemic therapy improved OS in patients with stage III-IV disease but not in those with stage I-II disease (Table 4 and Figure 4). The 2yOS (5yOS) was 96.0% (83.1%) and 94.1% (86.35%) in stage I-II disease (P = 0.3880) and 81.7% (66.2%) and 67.7% (58.0%) in stage III-IV disease (P = 0.0355), respectively, for patients receiving systemic therapy and those not receiving it (Figures 4A-4B). 

IMRT provides comparable efficacy to 3DCRT for the treatment of HPC (Table 2 and Table 4).

Toxicity

A total of 45 patients (26.3%) experienced acute adverse reactions of grade ≥ 3 (Table 5). Five patients (2.8%) experienced grade ≥ 3 late toxicities (Table 5). IMRT provides comparable safety to 3DCRT in the treatment of HPC not only in the acute phase (grade ≥ 3 toxicity: 18/87 =26.0% in 3DCRT and 27 in 84 = 32.1% in IMRT, P = 0.12), but also in the late phase (2/87 = 2.8% in 3DCRT and 3 /84 = 3.5% in IMRT, P = 0.678).

**Table 5:** Toxicity grade 3 or more. The sum of the patient numbers does not equal the total number because several patients experienced multiple grade 3 toxicities. *Leukopenia, neutropenia, including 2 febrile neutropenia **Hyperamylasemia ***including one radiation necrosis of the larynx

Acute Toxicity (N=45)	N	Late Toxicity (N=5)	N
Pharyngitis/dysphagia	28	Pharyngitis/ dysphagia***	4
Dermatitis	9	Aspiration pneumonia	1
Blood toxicity*	4	Dermatitis	1
Aspiration pneumonia	3	Osteomyelitis of the mandible	1
Liver dysfunction	2		
Renal dysfunction	1		
Other**	1		

## Discussion

This study aimed to evaluate the efficacy of radiotherapy for HPC, including the analysis of the impact of the new RT technique, IMRT, in comparison to the conventional RT technique, 3DCRT. To the best of our knowledge, this is one of the largest series of outcome reports on HPC treated with RT [2-6,11,13-16].

HPC is an aggressive form of cancer with the highest reported mortality rate among head and neck cancers [1-3]. Late clinical presentation is common, with 70-90% of patients presenting with Stage III or IV disease, including ours [1-3]. The estimated 5y OS for those receiving treatment is relatively poor at 5-65%, including ours, depending on tumor-related and patient-related factors, as well as treatment approaches (stage I: 30-65%; stage II: 30-55%; stage III: 10-40%; stage IV: 5-30%; JASTRO guideline 2024) [2-6,11,13-16]. Our data are comparable to the reported outcomes in the literature: the 5-year OS rates were 87.5%, 83.3%, 84.4%, and 59.3% for patients with stages I, II, III, and IV disease, respectively. As expected, our data revealed that the N category, which forms part of the staging classification and may be related to distant metastasis, is an important predictor of OS in multivariate analysis. In addition, the T category was an important predictor of LC and LRC in the univariate analysis, which is also plausible.

The management of HPC poses a formidable challenge in the field of head and neck oncology. As surgical management induces substantial functional morbidity in terms of speech and swallowing, alternative organ-preserving treatment modalities, particularly chemoradiotherapy-based approaches, are widely accepted as well-established strategies for preserving the larynx in surgically amenable HPC [2,3,11,-14,17-19]. In terms of overall survival, the EORTC 24981 trial established the non-inferiority of the IC-driven selective larynx preservation approach to the upfront surgical approach [7]. In addition, the RTOG 9111 trial showed no significant difference in OS among patients with advanced laryngeal cancer in the upfront surgery, upfront CCRT, and IC arms [8]. Subsequently, physicians extrapolated this treatment approach and its outcomes to the clinical standard, even though the trial did not include patients with HPC [2,3,11]. Standard drug therapy is high-dose, cisplatin-based chemotherapy [2,3,11]. If this is not tolerated, BRT with cetuximab [9]. TFP-based IC is also an option [7-8,2,3]. We could not identify any differences among the chemotherapy regimens.

Nutting et al. reported the reduction of the incidence of xerostomia, which led to recovery of saliva secretion and improvements in associated quality of life by IMRT in comparison to 3DCRT, and thus strongly support a role for IMRT in squamous-cell carcinoma of the head and neck [4]. Mok et al. reported that patients with HPC treated with IMRT showed a higher locoregional control compared with 3DCRT [5]. However, the distant relapse rate and OS remain comparable between the treatment techniques (5-mok). In contrast, Katsoulakis et al. reported that IMRT achieved LC and LRC rates comparable to those of conventional 3DCRT (6-Katsoulakis). Our data concur with the latter, showing that RT was equally effective as 3DCRT in terms of OS, LC, LRC, PFS, and toxicity. However, owing to the retrospective nature of the multi-institutional data collection, we could not obtain data on mild-to-moderate (grade 1-2) toxicities in the toxicity analysis. This result meant that we could not present a detailed IMRT toxicity profile, which may have overlooked the efficacy of IMRT in reducing toxicity. Therefore, although our data showed that 3D-CRT was as effective as IMRT and produced similar levels of toxicity, it cannot be applied outside Japanese institutions, given that variations in resources and techniques may exist elsewhere. A prospective randomized trial is required to confirm this equivalence.

In addition to the above, this study had some limitations. Multicenter data accumulation is prone to selection bias, which may compromise the completeness of the data and introduce heterogeneity in patients (detailed information on comorbidities, performance status, or HPV status was lacking), tumors, and treatment factors. In particular, the heterogeneous use of systemic therapy makes it difficult to interpret the role of each systemic agent. We hope that future prospective trials will clarify the potential of each systemic therapy regimen.

## Conclusions

Definitive radiotherapy achieved favorable OS, LC, LRC, and PFS in patients with HPC, with acceptable toxicity. IMRT provides comparable efficacy and safety to 3DCRT for the treatment of HPC.
